# Efficient One-Step Synthesis of a Pt-Free Zn_0.76_Co_0.24_S Counter Electrode for Dye-Sensitized Solar Cells and Its Versatile Application in Photoelectrochromic Devices

**DOI:** 10.3390/nano13202812

**Published:** 2023-10-23

**Authors:** Yerbolat Tashenov, Diana Suleimenova, Bakhytzhan Baptayev, Salimgerey Adilov, Mannix P. Balanay

**Affiliations:** 1 National Laboratory Astana, Nazarbayev University, 53 Kabanbay Batyr Ave., Astana 010000, Kazakhstan; tashenovyerbolat@gmail.com (Y.T.); www.lady.di@mail.ru (D.S.);; 2 Department of Chemistry, L.N. Gumilyov Eurasian National University, 2 Satpayev St., Astana 010008, Kazakhstan; 3 Department of Chemistry, Nazarbayev University, 53 Kabanbay Batyr Ave., Astana 010000, Kazakhstan

**Keywords:** ternary sulfide, dye-sensitized solar cell, Pt-free counter electrode, photoelectrochromic device, Prussian Blue

## Abstract

In this study, we synthesized a ternary transition metal sulfide, Zn_0.76_Co_0.24_S (ZCS-CE), using a one-step solvothermal method and explored its potential as a Pt-free counter electrode for dye-sensitized solar cells (DSSCs). Comprehensive investigations were conducted to characterize the structural, morphological, compositional, and electronic properties of the ZCS-CE electrode. These analyses utilized a range of techniques, including X-ray diffraction, scanning electron microscopy, energy dispersive X-ray spectroscopy, and X-ray photoelectron spectroscopy. The electrocatalytic performance of ZCS-CE for the reduction of I_3_^−^ species in a symmetrical cell configuration was evaluated through electrochemical impedance spectroscopy and cyclic voltammetry. Our findings reveal that ZCS-CE displayed superior electrocatalytic activity and stability when compared to platinum in I^−^/I_3_^−^ electrolyte systems. Furthermore, ZCS-CE-based DSSCs achieved power conversion efficiencies on par with their Pt-based counterparts. Additionally, we expanded the applicability of this material by successfully powering an electrochromic cell with ZCS-CE-based DSSCs. This work underscores the versatility of ZCS-CE and establishes it as an economically viable and environmentally friendly alternative to Pt-based counter electrodes in DSSCs and other applications requiring outstanding electrocatalytic performance.

## 1. Introduction

Dye-sensitized solar cells (DSSCs), referred to as the third generation of photovoltaic technology, have seen a surge in research efforts in recent decades. These innovative solar cells, first reported in 1991 and with a modest 7% efficiency, have since emerged as a promising alternative to expensive silicon-based photovoltaics. Their appeal lies in their ease of fabrication under ambient conditions, flexibility, and cost-effectiveness [1,2,3]. Recent advances in DSSCs have produced astonishing results. They achieve a remarkable energy conversion efficiency of 15.2% under standard 1.5 AM light conditions and an unprecedented PCE of 34% under ambient light conditions [4,5]. This outstanding performance positions DSSCs as viable options for outdoor and indoor applications [6,7]. Nevertheless, the commercialization of DSSCs has suffered setbacks primarily due to economic factors, including the cost of the technology and its moderate life expectancy. As a result, the focus of DSSC research has shifted to optimizing performance and reducing costs.

**Figure 1:**
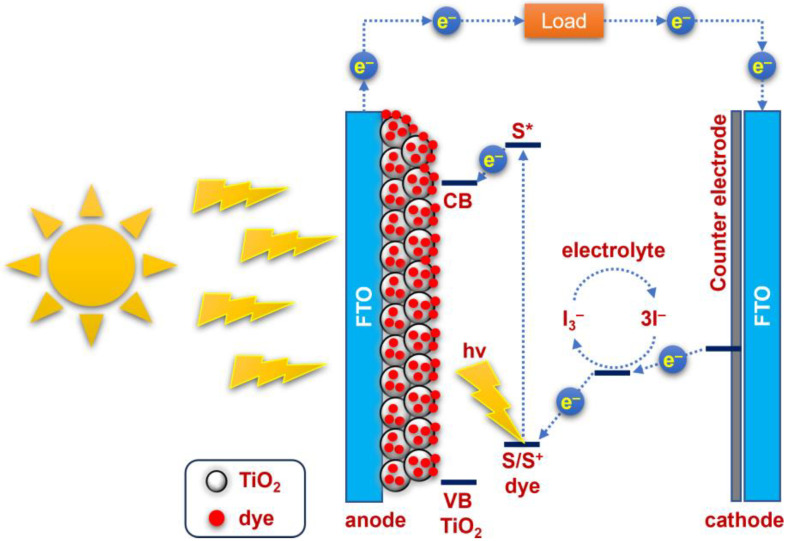
A schematic diagram of the operational principles of DSSC. CB and VB denote the conduction and valence band of the TiO_2_, respectively. S/S^+^ signifies the ground and cationic state of the dye, and S* represents the excited state of the dye. FTO stands for fluorine-doped SnO_2_.

Despite these challenges, the use of DSSCs represents an extremely novel and promising method for harnessing solar energy. The major components of these photovoltaic devices include the photoanode, sensitizers, electrolyte, and counter electrode, all of which are critical for effective operation. Figure 1 shows a schematic diagram illustrating the operation of DSSCs. The photoanode plays a central role in the absorption and conversion of solar energy. It is usually made of nanocrystalline titanium dioxide (TiO_2_), which is the preferred material due to its numerous advantageous properties, such as its excellent electron mobility, stability, and cost-effectiveness. Its compatibility with dye molecules due to its porosity and ease of fabrication make it a preferred choice for the efficient conversion of solar energy [8]. The sensitizers or dyes in DSSCs are responsible for capturing solar energy and converting it into electricity by absorbing light, generating electron–hole pairs, injecting electrons into the semiconductor, and facilitating regeneration of the sensitizer molecule to sustain the photovoltaic process. This mechanism enables DSSCs to efficiently harness sunlight and convert it into usable electrical energy. Among commercial dyes, N719 is still widely accepted as a reference dye, mainly because of its well-documented optical and electrochemical properties. In addition, it is preferred because of its relatively high conversion efficiency and standardized test protocols that allow researchers to evaluate the performance of DSSCs using established methods [9]. To enable the continuous conversion of absorbed sunlight into electricity in a DSSC, an essential component is the electrolyte solution located between the photoanode and the counter electrode. Among the most widely used and established electrolyte systems employed in DSSCs is the iodide/triiodide redox couple. This choice is mainly due to its exceptional efficiency in facilitating electron transfer and the regeneration of dye molecules [10]. In addition to traditional liquid electrolytes, the DSSC research community has recently shown increased interest in quasi-solid and solid-state electrolytes. These emerging alternatives have distinct advantages over their liquid counterparts, such as higher stability and lower potential for leakage. They are typically made from polymer or gel matrices, highlighting their potential for future advances in DSSC technology [11].

Due to its excellent catalytic activity, platinum is the conventional and widely accepted choice for counter electrodes (CEs) in DSSCs [12]. Pt enables efficient electron transport at the counter electrode and supports the regeneration of oxidized dye molecules. Moreover, platinum effectively drives the reduction of oxidized species in the electrolyte due to its low overpotential for the reduction reaction. However, one of the drawbacks of using Pt as a counter electrode is its high cost, which limits the scalability and affordability of DSSCs. In addition, Pt can be degraded and oxidized by the liquid electrolyte, especially when the redox pair I^−^/I_3_^−^ is used, leading to stability problems [13]. Recently, extensive efforts have been made to develop platinum-free counter electrodes. The ideal material should not only be inexpensive, but also have excellent electrocatalytic activity in the reduction of triiodide and high stability in corrosive environments. Among the various materials studied, transition metal chalcogenides stand out due to their impressive electrocatalytic activity, chemical stability, and abundant availability [14,15,16,17,18,19,20,21]. However, compared to Pt-based solar cells, DSSCs with transition metal chalcogenides still lag behind in terms of power conversion efficiency (PCE). In this context, ternary transition metal chalcogenides prove to be a more promising route. They are characterized by the presence of two cations that cause charge delocalization through d–d overlap within the crystal lattice. This phenomenon enhances the electrocatalytic activity of the material [22,23]. Recent studies have investigated counter electrodes based on ternary transition metal chalcogenides for DSSCs and obtained remarkable results, such as CoSbS (5.38%, Pt: 5.54%) [24], NiCo_2_S_4_ (8.29%, Pt: 7.35%) [25], CuCo_2_S_4_ (7.56%, Pt: 7.42%) [17], Ni_0.95_Mo_0.05_S (7.15%, Pt: 7.20%) [26], and NiAl_2_S_4_ (5.02%, Pt: 5.38%) [27]. In 2019, Li and Jin et al. presented a ternary sulfide counter electrode based on Zn_0.76_Co_0.24_S for DSSCs, which achieved a PCE of 7.42% using a two-step preparation method [28].

In this study, we introduce a Zn_0.76_Co_0.24_S-based DSSC counter electrode, designated as ZCS-CE, synthesized via a cost-effective one-step solvothermal process. ZCS-CE exhibits comparable photovoltaic performance to Pt-based DSSCs. Unlike prior research [28], which employed a more complex and expensive two-step synthesis method, we propose an innovative approach to enhance the catalytic activity of Zn_0.76_Co_0.24_S through a streamlined one-step process, reducing production costs. We comprehensively investigate its electrochemical behavior and conduct a comparative evaluation of composite DSSCs utilizing platinum and ZCS-CE as counter electrodes. This analysis reveals the superior durability of solar cells equipped with ZCS-CE, while also highlighting the cost-saving benefits of the one-step synthesis. The outcomes of this study hold the potential to advance DSSC technology by offering a more sustainable, eco-friendly, and economically viable approach to harness solar energy.

In the following sections, we will discuss the synthesis procedure, structural and morphological characterization, and electrochemical behavior of the Zn_0.76_Co_0.24_S electrocatalyst, and present a performance evaluation of the ZCS-CE in DSSCs. These results open new opportunities for the development of low-cost, environmentally friendly, and high-performance solar cells and contribute to the global efforts to harness solar energy for a clean and sustainable future. In addition to our primary focus on developing efficient and cost-effective DSSCs, we are expanding our research to explore potential applications of DSSCs in photoelectrochromic devices (PECDs). PECDs are devices that can dynamically change their optical properties in response to external stimuli such as light or applied voltage. The integration of DSSCs into PECDs holds promise for the development of smart and energy-efficient solutions in a variety of areas, including smart windows, eyeglasses, and various applications where adjustable transparency or coloration is desired.

## 2. Materials and Methods

### 2.1. Materials

All chemical reagents and materials are from commercial sources and have been used without further modification unless otherwise noted.

### 2.2. Synthesis of ZCS-CE 

A solution-based method was used to deposit a Zn_0.76_Co_0.24_S layer on a glass substrate coated with fluorine-doped tin oxide (FTO). Before depositing the ZCS-CE layer, the FTO substrates were thoroughly cleaned by washing with soapy water, deionized water, and acetone under ultrasonic conditions. To prepare the zinc–cobalt sulfide material by solvothermal synthesis, the following steps were performed: thiourea (0.5 mmol), Zn(NO_3_)_2_ × 6H_2_O (0.125 mmol), and Co(NO_3_)_2_ × 6H_2_O (0.250 mmol) were dissolved in 35 mL ethanol at room temperature for 30 min. This solution was then placed in a 50 mL stainless steel reactor with a Teflon liner, where the FTO glass substrate was attached to the wall with the conducting side down. The mixture was gradually heated at a rate of 5 °C per minute until it reached 200 °C, and this temperature was maintained for 4 h. After natural cooling to room temperature, the electrodes were repeatedly rinsed with deionized water and ethanol to remove unreacted compounds. They were then dried in a vacuum oven at 80 °C for 12 h. For comparison with the fabricated ZCS-CE, a control CE was also prepared by applying Pt paste (consisting of terpineol and hexachloroplatinic acid, Sigma-Aldrich, St. Louis, MO, USA) to a clean and dry FTO glass substrate and then annealing at 500 °C for 30 min.

### 2.3. Fabrication of Dye-Sensitized Solar Cells

Preparation of the photoanode began with cutting FTO glass (2.2 mm, surface resistivity 7 Ω/sq, Sigma-Aldrich) into pieces of 1.5 × 2.0 cm, washing in ethanol and acetone for 30 min each with ultrasound, and then drying under reduced pressure. A compact TiO_2_ film was deposited by immersing the clean and dry FTO slides in a 50 mM titanium (IV) isopropoxide solution in 1 M aqueous HCl for 30 min and then sintering at 500 °C for the same time. The compact film was then coated with a transparent TiO_2_ paste (DN-EP03, Dyenamo (Stockholm, Sweden), particle size: 18–20 nm) using a doctor blade technique. The film was air-dried for one hour before being sintered stepwise at 125 °C, 325 °C, 425 °C, and 500 °C. The transparent TiO_2_ film was coated with a scattering TiO_2_ film (Greatcell Solar WER2-O, Sigma-Aldrich, particle size: 150–250 nm) by doctor blading after cooling to 70 °C. The film was then air-dried for one hour before being sintered in the same manner as the transparent TiO_2_ layer. The electrodes were then immersed in a solution of 0.25 mM dye N719 (Sigma-Aldrich) in a mixture of acetonitrile:tert-butanol (1:1) with 0.75 mM chenodeoxycholic acid (CDCA, Sigma-Aldrich) for 24 h. The dye-loaded electrodes were then carefully washed with ethanol before drying. Finally, the counter electrode was securely attached to the TiO_2_ photoanode with double-sided tapes acting as spacers after dropping the iodide/triiodide redox electrolyte (DN-OD03 S104, Dyenamo) over it.

### 2.4. Deposition of Prussian Blue and Fabrication of Photoelectrochromic Device

After ultrasonic cleaning in a mixture of ethanol and acetone for 15 min, a glass substrate coated with FTO with a size of 0.5 × 1.5 cm^2^ was used as a conductive substrate for the Prussian Blue (PB) electrochromic layer. This electrochromic layer was deposited on the FTO surface using the electrodeposition method according to a procedure adopted from the literature [29] with minor modifications. Specifically, the PB film was created using the chronoamperometry method with a deposition time of 5 s and a voltage of 0.4 V. A standard three-electrode configuration was used for this deposition process, with the FTO-coated glass, Ag/Ag^+^, and Pt wires serving as working, reference, and counter electrodes, respectively. The electrolyte solution consisted of 20 mM each of HNO_3_, NaNO_3_, Fe(NO_3_)_3_ × 9H_2_O, and K_3_[Fe(CN)_6_] in a 30 mL electrodeposition bath.

In the fabrication of the photoelectrochromic device, two prefabricated dye-sensitized solar cells were connected in series with a Pt wire. In addition, a PB-coated FTO substrate was immersed in an electrolyte solution. The anode (−) of the DSSCs was connected to the PB-coated FTO, while the ZCS-CE-based counter electrode (+) was connected to the Pt wire via an outer conductor. The electrolyte solution contained 0.1 M NaNO_3_ and 0.1 M HNO_3_. The performance of the photoelectrochromic device was investigated under sunlight irradiation (AM 1.5).

### 2.5. Characterization

An X-ray diffractometer (XRD, Rigaku SmartLab X-ray, Cedar Park, TX, USA) was used to determine the structural features of the synthesized materials. Energy dispersive X-ray spectroscopy and NEXSA Thermo Scientific (Waltham, MA, USA) X-ray photoelectron spectroscopy (XPS) were used to determine the material composition, while a Zeiss (Jena, Germany) Crossbeam 540 scanning electron microscope (SEM) was used to study the morphological properties. The photovoltaic evaluation was performed using the Dyenamo toolbox (DN-AE01). A Palmsens4 potentiostat was used for electrochemical experiments. The electrochemical impedance spectroscopy (EIS) of the DSSCs was performed in complete darkness with a bias voltage of −0.72 V, an amplitude of 10 mV, and a frequency range of 0.1–100,000 Hz. EIS was performed on the symmetrical dummy cells under the same conditions but with a bias voltage of 0 V. Cyclic voltammetry (CV) to evaluate the catalytic activity of the electrocatalysts was performed at a scan rate of 0.1 V/s using Ag/Ag^+^, a Pt wire, and the prepared materials (Pt and/or ZCS-CE films on FTO glass) with the working area of 2.25 cm^2^ as the reference electrode, CE, and working electrode, respectively. LiI (10 mM), LiClO_4_ (0.1 M), and I_2_ (1 mM) solution in acetonitrile (50 mL) were the components of the electrolyte used for the CV test. A Thermo Scientific Evolution 300 UV-Vis spectrophotometer was used to study the optical performance of the electrochromic device (ECD). The experiment to evaluate the ECD was performed in a three-electrode system using Prussian Blue film on FTO glass as the working electrode, platinum wire as the counter electrode, and Ag/Ag^+^ as the reference electrode. Cycles were performed with an aqueous electrolyte of 0.1 M NaNO_3_ and 0.1 M HNO_3_ (30 mL) and within a potential range of −0.5 V to +1.0 V. The working area of the PB film was 0.5 cm^2^ and the voltage scan rate was 20 mV/s.

## 3. Results and Discussion

### 3.1. Characterization of ZCS-CE

**Figure 2:**
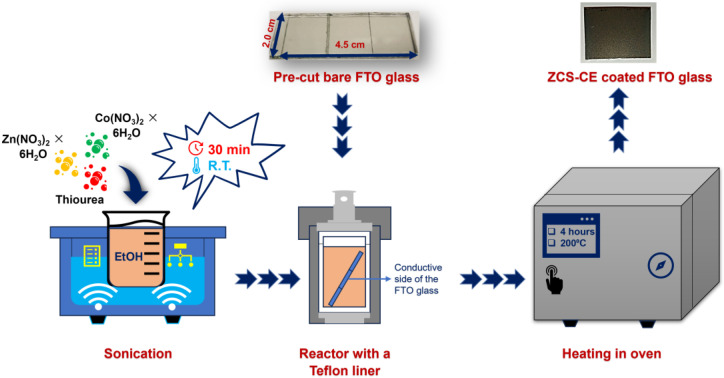
Schematic illustration of the one-pot synthesis of a Zn_0.76_Co_0.24_S counter electrode on an FTO substrate.

Ternary zinc–cobalt sulfide was synthesized using a solvothermal one-pot method, as shown in Figure 2. The precursors of the elements Zn, Co, and S were taken in suitable molar ratios and thoroughly dissolved by stirring and sonication. Heating the prepared solution at 200 °C for 4 h together with a glass FTO substrate in a Teflon-lined autoclave reactor resulted in the formation of a ZCS-CE film on the FTO.

**Figure 3:**
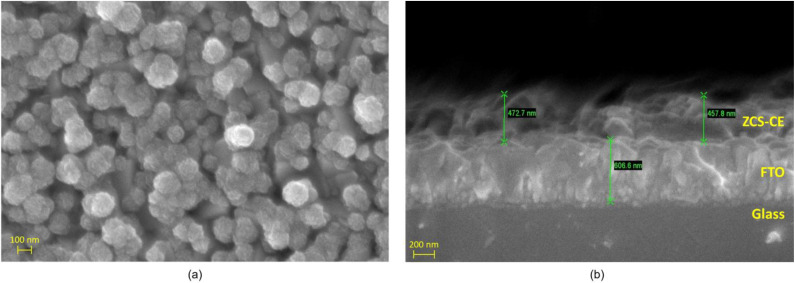
Scanning electron microscope images of the ZCS-CE: (**a**) top view and (**b**) cross-sectional perspective.

The morphological characteristics of the ZCS-CE sample prepared on the FTO substrate were analyzed using scanning electron microscopy (see Figure 3). As shown in Figure 3a, the ZCS-CE has a predominantly spherical shape with diameters between 100 and 150 nm. The SEM image shows the presence of microspheres, each of which has a distinct nanoflock-like structure and forms a recognizable cluster on the surface. Figure 3b provides an insight into the thickness of the ZCS-CE layer on the FTO glass, which measures approximately 465 nm. This image shows a smooth, flat, and uniform growth of the ZCS-CE layer that is consistent along its entire length.

**Figure 4:**
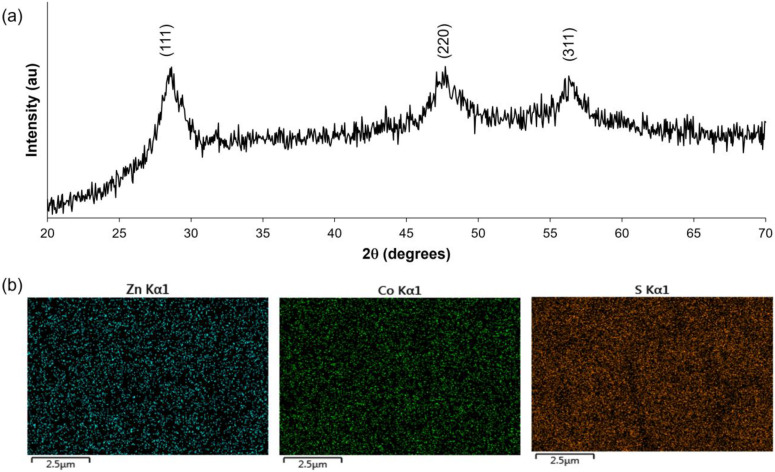
(**a**) XRD spectrum revealing crystallographic information and (**b**) elemental mapping of Zn, Co, and S distribution of the of as-prepared ZCS-CE.

The as-prepared ZCS-CE sample underwent thorough investigation by powder X-ray diffraction analysis to confirm its purity and crystal structure. In Figure 4a, the X-ray diffraction pattern of ZCS-CE shows characteristic peaks at 2θ-diffraction angles of 28.6°, 47.6°, and 56.4° corresponding to the (111)-, (220)-, and (311)-planes of the cubic phase of Zn_0.76_Co_0.24_S (JCPDS #47-1656) [30]. The pronounced sharpness of these peaks and the conspicuous absence of extraneous signals in the diffraction pattern are compelling evidence of the high crystallinity and impeccable purity of the ZCS-CE material. In particular, these results confirm the successful synthesis of ZCS-CE by a simple one-step solvothermal process. To validate the presence and uniform distribution of the constituent elements (Zn, Co, and S) on the FTO glass substrate, we employed energy dispersive spectroscopy (EDS), as depicted in Figure 4b. EDS is a powerful technique that not only identifies the elemental composition of materials but also provides spatial information about their distribution. The EDS analysis reaffirms the presence of all expected elements throughout the examined area, and equally crucially, it demonstrates their uniform dispersion. This uniformity is of paramount importance in various applications, notably in the development of semiconductor materials and photovoltaic devices. In these contexts, the consistency of element distribution significantly influences material performance and efficiency.

**Figure 5:**
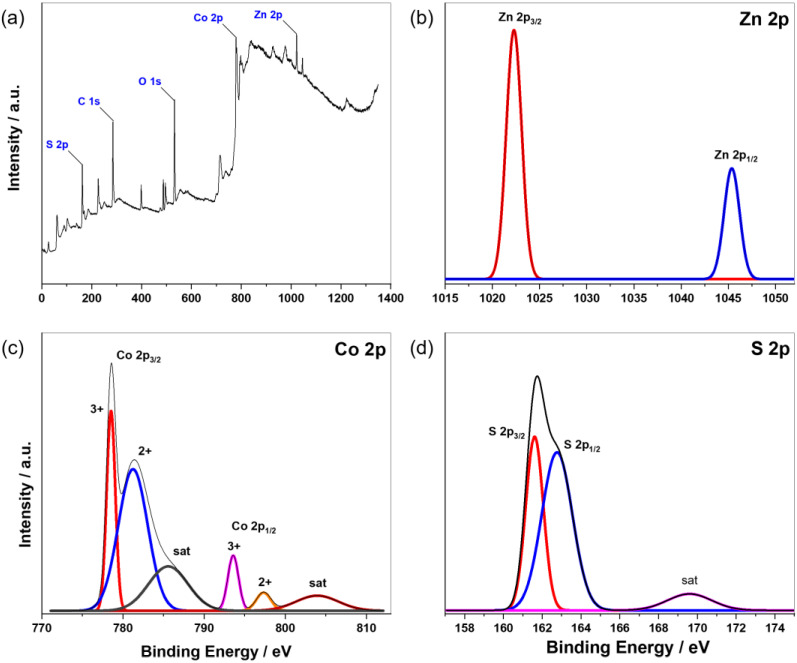
(**a**) XPS survey spectrum of the as-synthesized ZCS-CE. High-resolution XPS spectra of (**b**) Zn 2p, (**c**) Co 2p, and (**d**) S 2p.

X-ray photoelectron spectroscopy was employed to investigate the chemical composition and oxidation states of the elements. In Figure 5a, the XPS spectrum reveals characteristic peaks corresponding to the elements Zn, Co, and S, all possessing a 2p electron configuration. These findings align with the results of energy dispersion X-ray spectroscopy. Notably, the presence of carbon and oxygen peaks can be attributed to the adsorption of CO_2_ and moisture from ambient air on the sample’s surface. For a more in-depth examination of the chemical states, we turn to the high-resolution spectrum of Zn 2p (Figure 5b), in which two prominent peaks at 1022.3 eV and 1045.4 eV correspond to Zn 2p_3/2_ and Zn 2p_1/2_, respectively. These peaks confirm the 2+ oxidation state of zinc [31]. Similarly, the Co 2p emission spectrum in Figure 5c displays the coexistence of 2+ and 3+ oxidation states at the surface of the ZCS-CE sample. Here, two primary peaks at 781.2 eV (Co 2p_3/2_) and 797.3 eV (Co 2p_1/2_) are associated with Co^2+^, while two additional sharp peaks at 778.5 eV and 793.6 eV represent Co^3+^, confirming their distinct oxidation states. The presence of Co 2p satellite peaks at 785.6 eV and 803.9 eV supports these results [32]. The high-resolution S 2p spectrum, shown in Figure 5d, exhibits an asymmetric peak that can be divided into two distinct peaks at 161.6 eV and 162.8 eV, corresponding to S 2p_3/2_ and S 2p_1/2_, respectively. These peaks align with metal–sulfur bonds as described in the literature [33]. Additionally, a broad satellite peak at 169.6 eV suggests various forms of oxidized sulfur. Overall, the XPS analysis conclusively establishes the chemical composition of the ZCS-CE sample, including the Zn^2+^, Co^2+^, Co^3+^, and S^2−^ states, which agrees seamlessly with the XRD results.

### 3.2. Electrochemical Analysis of ZCS-CE

**Figure 6:**
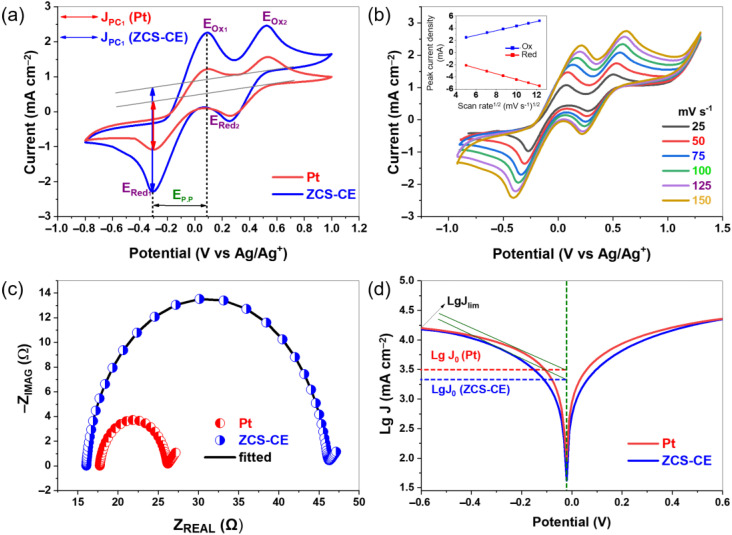
(**a**) Cyclic voltammograms of the counter electrodes; (**b**) cyclic voltammetry at various scan rates of ZCS-CE; (**c**) Nyquist plot of symmetrical dummy cells; and (**d**) Tafel plots of counter electrodes.

To evaluate the electrocatalytic performance of our fabricated ZCS-CE, cyclic voltammetry experiments were performed using a standard three-electrode configuration and a scan rate of 100 mV/s. As shown in Figure 6a, both the Pt and ZCS CEs show distinct anodic and cathodic peaks associated with two electron transfer processes involving iodide/triiodide redox reactions. These reactions can be described by Equations (1)–(4), which represent the oxidation and reduction of I_3_^−^/I_2_ and I^−^/I_3_^−^ species at the electrode surface.

(1) $${\mathrm{Ox}}_{1}\text{:}\;3I^{-}\to I_{3}^{-}+2e^{-}$$

(2) $${\mathrm{Ox}}_{2}\text{:}\;2{I_{3}}^{-}\to 3I_{2}+2e^{-}$$

(3) $${\mathrm{Red}}_{1}\text{:}\;{I_{3}}^{-}+2e^{-}\to 3I^{-}$$

(4) $${\mathrm{Red}}_{2}\text{:}\;3I_{2}+2e^{-}\to 2I_{3}^{-}$$

Usually, the electrocatalytic performance of CE materials is evaluated in terms of I_3_^−^ reduction using two key parameters derived from the left pair of oxidation-reduction peaks (Ox_1_/Red_1_): the peak cathodic current density (J_PC1_) and the peak-to-peak distance (E_p.p_) between the Ox_1_ and Red_1_ peaks. Higher J_PC_ values and lower E_p.p_ values for the counter electrodes indicate lower overpotentials for the initiation of the I^−^/I_3_^−^ redox reaction and higher reaction rates, respectively. In our study, the ZCS-CE outperforms its Pt counterpart by exhibiting higher peak cathodic current density. This result indicates that triiodide is reduced faster on the ZCS-CE surface, suggesting better overall electrocatalytic activity. Moreover, both the Pt and ZCS CEs exhibit the same E_p.p_ value (0.38 V), indicating their comparable abilities to initiate the I^−^/I_3_^−^ redox reaction. Figure 6b shows the CV of ZCS-CE at different scan rates, with the redox peak signals becoming more intense at higher scan rates. The linear relationship between the current density of the redox peaks and the square root of the scan rate (Figure S1, Supporting Information) shows that the electrocatalyst and the redox electrolyte do not interact chemically. Instead, ion diffusion in particular facilitates the reduction of triiodide.

Electrochemical impedance spectroscopy was used to evaluate the electrocatalytic activity of the ZCS-CE in symmetrical dummy cells with a CE/electrolyte/CE configuration. The Nyquist plots for both the ZCS-CE and Pt were fitted using the electrical equivalent circuit based on the Randles model (see Figure S2 in the Supporting Information), resulting in well-defined semicircles, as presented in Figure 6c. The series resistance (R_s_) of the electrodes, measured at 16.05 Ω for ZCS-CE and 17.72 Ω for Pt, marks the starting point of these semicircles in the Nyquist diagram. These results indicate excellent adhesion and contact behavior between the FTO substrate and the ZCS-CE electrocatalyst, with lower ohmic resistance compared to Pt. The semicircular region provides information about the charge transfer resistance (R_CT_) at the interface between the electrocatalyst and the electrolyte. In general, a smaller R_CT_ value indicates higher catalytic activity in the reduction of I_3_^−^/I^−^ during charge transfer. From the EIS–Nyquist plots, the R_CT_ values for the ZCS-CE and Pt catalysts are 14.97 Ω cm^2^ and 4.18 Ω cm^2^, respectively. These results highlight the increased internal resistance at the electrode–electrolyte interface and the reduced electrocatalytic reduction of triiodide ions by the ZCS-CE electrocatalyst compared to the reference electrode, which ultimately affects the performance of the DSSCs.

Tafel polarization curves were employed to evaluate the catalytic performance of the CE materials, as shown in Figure 6d. The Tafel polarization curve describes three distinct regions—the polarization region, the Tafel region, and the diffusion region—based on different voltage levels, from low to high. The polarization region is located in the lower potential region, the Tafel region is in the middle and plays a central role in controlling the electrocatalytic activity, while the diffusion region covers the rest of the curve and determines the diffusion behavior of the cathode ions. Two critical parameters, namely the limiting diffusion current (J_lim_) and the exchange current density (J_0_), can be derived from the Tafel profiles. These parameters are critical for evaluating the electrocatalytic performance of the CE. As shown in Figure 6d, the exchange current density within the Tafel region can be determined by identifying the intersection of the slope with the zero voltage. In addition, Equation (5) provides an alternative method for calculating J_0_, as follows:

J_0_ = RT/nFR_CT_ (5)

In this equation, R represents the universal gas constant, T represents the absolute temperature, n represents the number of electrons transferred during the redox process, F represents the Faraday constant, and R_CT_ is derived from the EIS experiment. In EIS measurements, high catalytic activity was observed at low RCT values, which should translate into high J_0_ values according to Equation (5). When we compared the ZCS-CE with the Pt, we found that both have a slope in the range of log J_0_ = 3.30–3.50 mA/cm^2^, which indicates comparable catalytic activity and is consistent with the results of EIS measurements. At the same time, the high value of J_lim_ indicates a fast ion diffusion rate within the electrocatalyst. Remarkably, both ZCS-CE and Pt have almost identical J_lim_ values on the panel curves, indicating that the I^−^ and I_3_^−^ ions on the surfaces of both electrodes have similar diffusion rates, which affects the power conversion efficiency values of the dye-sensitized solar cells.

### 3.3. Photovoltaic Performance of ZCS-CE

**Figure 7:**
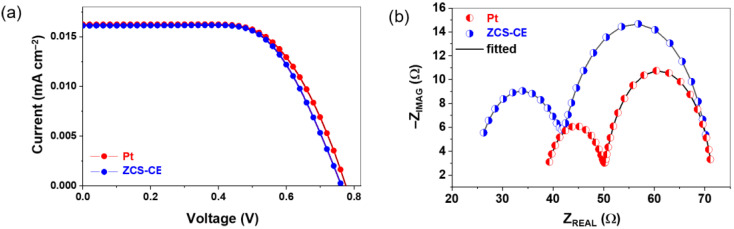
(**a**) Current–voltage curves and (**b**) Nyquist plot of DSSCs with Pt and ZCS CEs counter electrodes.

The current–voltage (J-V) characteristics of the newly fabricated dye-sensitized solar cells with ZCS-CE and standard Pt counter electrodes were measured in order to evaluate and compare their photovoltaic performance (see Figure 7a). In Table 1, the resulting photovoltaic parameters are summarized, providing values for the open-circuit voltage (V_OC_), short-circuit photocurrent density (J_SC_), fill factor (FF), and power conversion efficiency. 

The DSSC with a Pt counter electrode showed remarkable photovoltaic performance with V_OC_, J_SC_, and FF values of 0.780 V, 16.24 mA/cm², and 0.64, respectively, resulting in a PCE of 8.12%. By contrast, the DSSC with the ZCS-CE counter electrode achieved slightly lower performance with V_OC_ = 0.760 V, J_SC_ = 16.12 mA/cm², and FF = 0.65, corresponding to an efficiency of 7.94%. Although the photovoltaic properties of the ternary sulfide-based counter electrode lag behind those of the Pt counter electrode, they remain remarkably comparable, with only 2.2% difference from the reference electrode but with a much lower production cost.

The superior catalytic activity of the Pt layer contributed significantly to the higher J_SC_ value observed for the prepared DSSC, which is consistent with our results from Tafel measurements. The lower open circuit voltage values measured for the DSSCs with the ZCS-CE counter electrode corresponded to a higher charge transfer resistance at the interface between the electrocatalyst and the electrolyte, which is consistent with our electrochemical impedance spectroscopy results from the symmetrical dummy cells. To properly account for the impact of ZCS-CE on the electrochemical performance of DSSCs, we performed EIS on a complete cell and fitted the plots shown in Figure 7b with the equivalent electrical circuit presented in Figure S3 (Supporting Information). The EIS analysis yielded critical electrochemical insights, including the charge transfer resistance (R_CT_*) at the counter electrode–electrolyte interface (represented by a small semicircle on the EIS plot), the resistance at the TiO_2_–dye–electrolyte interface ($R_{TiO_{2}}$, a large semicircle), and the series resistance (R_S_*, where the curve crosses the abscissa axis). These parameters are detailed in Table 1. The electrocatalytic activity is directly reflected by the size of the small semicircle, correlating with R_CT_* at the counter electrode–electrolyte interface. Generally, a lower R_CT_* value signifies heightened electrocatalytic activity in counter electrode materials for redox reactions within the electrolyte. When comparing the two counter electrodes, the Pt-based DSSC exhibited a lower R_CT_* value at 11.83 Ω, while the solar cell employing the ZCS-CE counter electrode demonstrated a higher charge transfer resistance of 17.63 Ω, which is consistent with our previous results with the symmetrical dummy cells. 

**Figure 8:**
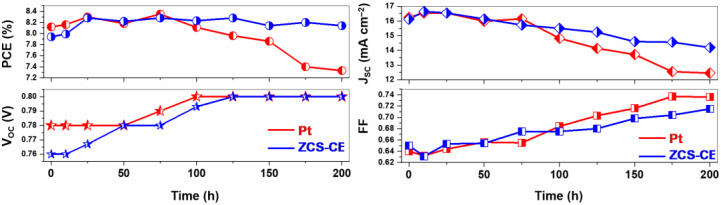
Photovoltaic stability of the different CEs used in DSSCs.

Additionally, we assessed the stability of DSSC devices fabricated with both ZCS-CE and Pt counter electrodes at room temperature. Photovoltaic parameters were periodically monitored for up to 200 h (Figure 8). The observed J_SC_, V_OC_, FF, and PCE values were recorded by averaging data from three devices. Notably, the stability of the Pt-based DSSC exhibited fluctuations within the first 100 h, peaking at a PCE value of 8.35% at the 75 h mark while maintaining a consistent efficiency of 8.11%. However, the power conversion efficiency for the Pt counter electrode began to decline after 100 h, eventually reaching 7.33% by the end of the stability test. This decline may be attributed to the deactivation of Pt’s catalytic properties upon extended exposure to the corrosive I_3_^−^/I^−^ electrolyte. Conversely, DSSCs utilizing the ternary sulfide counter electrode displayed superior stability. Their PCE steadily increased during the initial 25 h, achieving its highest efficiency peak of 8.28% at both the 25 h and 75 h marks. At the conclusion of the experiment, the solar cell equipped with the ZCS-CE maintained an efficiency of 8.14%, surpassing the standard platinum analog’s performance by 10% over the same timeframe. Notably, this difference was primarily driven by the J_SC_ value, as the V_OC_ values for both Pt and ZCS CEs remained constant after 100 h, reaching a voltage plateau at 0.8 V. These stability test results underscore ZCS-CE as an ideal counter electrode material for dye-sensitive solar cells.

In a recent study by the research team of Li and Jin [28], Zn_0.76_Co_0.24_S was synthesized in a two-step process. This process first involved the hydrothermal synthesis of a transition metal oxide, ZnCo_2_O_4_, followed by its conversion to sulfide using thioacetamide as a sulfur source. By contrast, our one-step technique provides a leaner and more efficient route to the production of Zn_0.76_Co_0.24_S, which serves as a promising counter electrode material. Our approach has several notable advantages, especially in terms of photovoltaic conversion efficiency and open circuit voltage. Our solar cells based on Zn_0.76_Co_0.24_S achieved an efficiency of 7.94%, exceeding the 7.42% reported in the reference study. In addition, our cells exhibited a higher V_OC_ of 0.76 V compared to the 0.65 V, indicating the potential for improved efficiency and higher output voltage. However, it is worth noting that the reference showed better current generation, with a higher short-circuit current of 17.5 mA/cm^2^ compared to our 16.12 mA/cm^2^. This discrepancy can be attributed to differences in the size of the nanoparticles. Their particles had a diameter of about 500 nm, while our synthesis resulted in particles with a diameter of 100 to 150 nm. Unfortunately, their studies did not provide information on the thickness of their counter electrode or the concentration of the N719 dye used, which could also affect photovoltaic performance. Overall, our one-step approach not only improves the efficiency of Zn_0.76_Co_0.24_S synthesis, but also provides a more cost-effective and easily scalable process for the fabrication of high-performance solar cells.

### 3.4. Fabrication and Characterization of Photoelectrochromic Device

**Figure 9:**
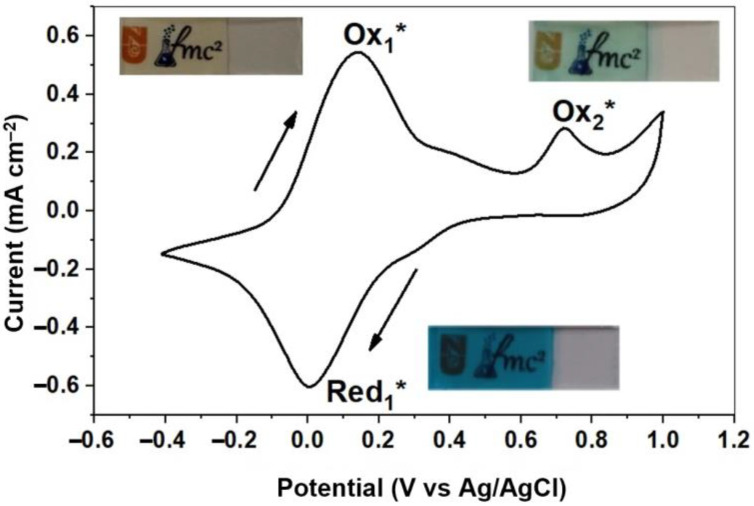
Electrochemical behavior of the Prussian Blue layer within the voltage range of −0.5 V to 1.0 V. The insets illustrate the visual color changes observed at various electrochemical states.

To comprehensively evaluate the electrochemical properties of thin Prussian Blue films, cyclic voltammetry measurements were performed at a scan rate of 20 mV/s. A three-electrode system was used with a solution containing 0.1 M NaNO_3_ and 0.1 M HNO_3_. The potential range investigated was from −0.5 V to 1.0 V. Figure 9 illustrates the resulting cyclic voltammogram.

The voltammogram shows two detectable oxidation peaks and one reduction peak, indicating the presence of two relatively stable redox couples within the system as well as an additional oxidation process. Consistent with the chemical reactions described below, the observed redox peak in the −0.4–0.4 V range corresponds to the conversion of Prussian Blue to Prussian White (PW) [34]. This transformation is driven by the reduction of Fe^3+^ ions, accompanied by the intercalation of sodium ions:

(6) $${{\mathrm{Red}}_{1}}^{*}\text{:}\;{\mathrm{KFe}}^{\mathrm{III}}[{\mathrm{Fe}}^{\mathrm{II}}(\mathrm{CN}{)}_{6}]+{\mathrm{Na}}^{+}+e^{-}\to {\mathrm{KNaFe}}^{\mathrm{II}}[{\mathrm{Fe}}^{\mathrm{II}}(\mathrm{CN}{)}_{6}]$$

Conversely, the reverse oxidation process together with the deintercalation of sodium ions leads to coloration:

(7) $${{\mathrm{Ox}}_{1}}^{*}\text{:}\;{\mathrm{KNaFe}}^{\mathrm{II}}[{\mathrm{Fe}}^{\mathrm{II}}(\mathrm{CN}{)}_{6}]\to {\mathrm{KFe}}^{\mathrm{III}}[{\mathrm{Fe}}^{\mathrm{II}}(\mathrm{CN}{)}_{6}]+{\mathrm{Na}}^{+}+e^{-}$$

The second oxidation peak at 0.72 V (145.24 μA) can be attributed to the partial oxidation of Fe^2+^ ions in PB:

(8) $${{\mathrm{Ox}}_{2}}^{*}\text{:}\;{\mathrm{KFe}}^{\mathrm{III}}[{\mathrm{Fe}}^{\mathrm{II}}(\mathrm{CN}{)}_{6}]\to {\mathrm{Fe}}^{\mathrm{III}}[{\mathrm{Fe}}^{\mathrm{III}}(\mathrm{CN}{)}_{6}]+K^{+}+e^{-}$$

**Figure 10:**
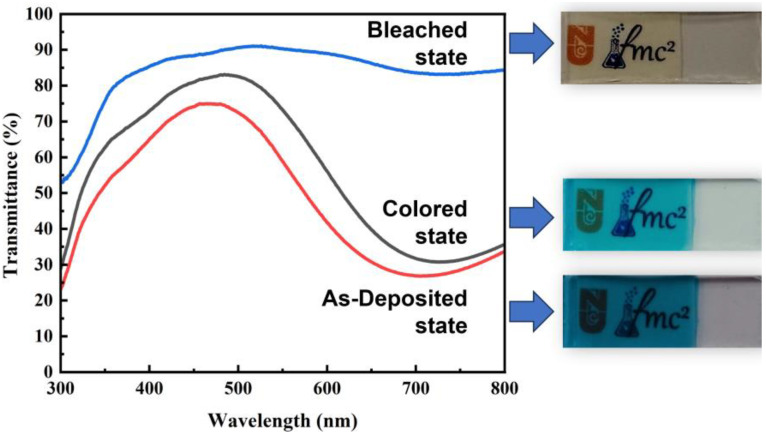
Transmittance spectra of Prussian Blue in its bleached, colored, and as-deposited states, accompanied by visual images corresponding to each state.

The optical properties of the PB films were thoroughly investigated using transmittance spectroscopy, which covers a wide wavelength range from 300 to 800 nm. Figure 10 shows the transmittance spectra of PB in its original, bleached, and dyed states. From these spectra, we extracted critical data points, highlighting the maximum transmittance at 488 nm and the minimum at 726 nm. To comprehensively evaluate the performance of these coatings, we used key parameters such as electrochromic contrast (ΔT), contrast ratio (CR), and optical density (ΔOD), which were calculated according to Equations (9)–(11). These crucial parameters are briefly summarized in Table 2. This rigorous analysis provides a comprehensive understanding of the optical properties and performance of PB films.

ΔT = T_bleached_ − T_colored_ (9)

CR = T_bleached_/T_colored_ (10)

ΔOD = log (CR)(11)

The parameters given vividly show the possibilities of optical modulation in the dye bleaching process. From Figure 10 and Table 2, it can be seen that the most significant transmittance shift of 52.49% and the highest ΔOD value of 0.43, which distinguish between the transparent and blue states, occur at a wavelength of 726 nm. Conversely, the lowest transmission shift of 7.12% and the lowest ΔOD value of 0.03, which distinguish between the bleached and colored states, are mainly observed at 488 nm.

Recently, there has been increased interest in the development of smart windows that can be optically controlled, especially as part of green building initiatives [35,36,37]. Smart windows have attracted attention because of several advantages they offer over conventional window systems. These include potential savings in air conditioning, heating, lighting, and curtain requirements. Among the various technologies being explored for smart windows, photoelectrochromic devices have emerged as a promising solution. PECD can combine the principles of a dye-sensitized solar cell and an electrochromic device, resulting in a robust and reliable approach. These devices feature the remarkable ability to reversibly change their coloration or transparency in response to external stimuli. At the same time, the DSSCs integrated into the PECD efficiently convert incident light into electrical energy. This combination of technologies holds significant potential for achieving dynamic control over various aspects of window functionality. These integrated systems can seamlessly regulate light transmission, tinting, and energy generation to help promote sustainable and energy-efficient building practices.

**Figure 11:**
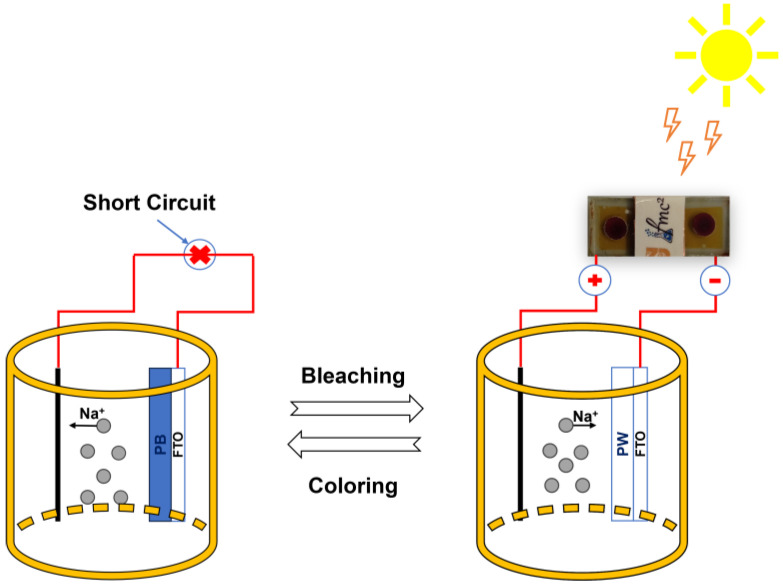
Schematic illustration of the photoelectrochromic device powered by dye-sensitized solar cells.

By using dye-sensitized solar cells as an energy source, we seamlessly integrated an electrochromic PB film into the design of a state-of-the-art photoelectrochromic device. The schematic diagram of our experimental electrochromic device is shown in Figure 11. In this configuration, we had two solar cells connected in series. The negative terminal of the DSSCs was connected to a PB-coated fluorine-doped tin oxide substrate, while the positive electrode was connected to another platinum (Pt)-coated FTO substrate via an outer conductor. The electrolyte solution used in this setup consisted of a 0.1 M NaNO_3_ + 0.1 M HNO_3_ mixture. Upon exposure to light, the electrochromic PB film transitioned from its initial state to a pale white state. This transition was reversible, and a short-circuit mechanism was used to return the electrochromic film to its original state, thereby restoring its coloration.

## 4. Conclusions

In summary, this study represents significant progress in the field of renewable energy and smart window technologies by synthesizing and characterizing Zn_0.76_Co_0.24_S, a ternary transition metal sulfide electrode prepared by a one-step solvothermal technique. When used as a counter electrode in dye-sensitized solar cells, ZCS-CE exhibits exceptional electrocatalytic activity with a competitive power conversion efficiency of 7.94%, making it a promising alternative to conventional platinum-based CEs. Comprehensive material analyses, including energy dispersive X-ray spectroscopy, X-ray photoelectron spectroscopy, X-ray diffraction, and scanning electron microscopy, confirmed the high crystallinity and purity of ZCS-CE and highlight its suitability for DSSC applications. In particular, ZCS-CE showed superior stability in electrolyte media and maintained its efficiency over 200 h of testing. Moreover, the integration of ZCS-CE into a photoelectrochromic device represents a novel, self-powered system that combines DSSC and electrochromic PB film technologies. This advancement holds great promise for smart window applications, enabling dynamic control of tint and transparency for sustainable and energy-efficient building design, shaping the future of renewable energy and architectural innovation.

## Figures and Tables

**Table 1:** Photovoltaic parameters and electrochemical impedance spectroscopy results of Pt- and ZCS-CE-based CEs.

CE	PCE (%)	V_OC_ (V)	J_SC_ (mA/cm^2^)	FF	R_CT_ (Ω cm^2^)	R_S_* (Ω)	R_CT_* (Ω)	$R_{TiO_{2}}$ (Ω)
ZCS-CE	7.94 ± 0.02	0.760 ± 0.00	16.12 ± 0.07	0.65 ± 0.00	14.97 ± 0.38	24.32 ± 0.77	17.63 ± 0.31	29.23 ± 0.29
Pt	8.12 ± 0.09	0.780 ± 0.00	16.24 ± 0.20	0.64 ± 0.02	4.18 ± 0.69	38.41 ± 0.64	11.83 ± 0.41	21.35 ± 0.31

**Table 2:** Transmittance (T), electrochromic contrast (ΔT), contrast ratio (CR), and optical density (ΔOD) of Prussian Blue in three different states.

λ (nm)	T_as-deposited_ (%)	T_colored_ (%)	T_bleached_ (%)	ΔT (%)	CR	ΔOD
488	74.11	83.03	90.15	7.12	1.08	0.03
726	27.18	30.74	83.23	52.49	2.71	0.43

## Data Availability

Data sharing not applicable.

## Supplementary Materials

The following supporting information can be downloaded at: https://www.mdpi.com/article/10.3390/nano13202812/s1, Figure S1: Correlation between peak current density and scan rate for Zn_0.76_Co_0.24_S; Figure S2: Equivalent electrical circuit used for fitting Nyquist plots of symmetrical dummy cells; Figure S3: Equivalent electrical circuit used for fitting Nyquist plots of DSSCs; Figure S4: Scanning electron microscopy images of PB film deposited on FTO glass at different resolutions.
