# Supplementary material for: Efficient One-Step Synthesis of a Pt-Free Zn0.76Co0.24S Counter Electrode for Dye-Sensitized Solar Cells and Its Versatile Application in Photoelectrochromic Devices
Source: Nanomaterials (Basel). 2023 Oct 23;13(20):2812. doi: 10.3390/nano13202812 (PMC10610264; doi:10.3390/nano13202812)
Supplement: Supplementary file 1 [file nanomaterials-13-02812-s001.zip › nanomaterials-2646221-supplementary.pdf]

Article

Efficient One-Step Synthesis of a Pt-Free $Zn_{0.76}Co_{0.24}S$ Counter Electrode for Dye-Sensitized Solar Cells and Its Versatile Application in Photoelectrochromic Devices

Yerbolat Tashenov [1,2], Diana Suleimenova [1,2], Bakhytzhan Baptayev [1], Salimgerey Adilov [1,3,*] and Mannix P. Balanay [1,3,*]

[1] National Laboratory Astana, Nazarbayev University, 53 Kabanbay Batyr Ave., Astana 010000, Kazakhstan; tashenovyerbolat@gmail.com (Y.T.); www.lady.di@mail.ru (D.S.)
[2] Department of Chemistry, L.N. Gumilyov Eurasian National University, 2 Satpayev St., Astana 010008, Kazakhstan
[3] Department of Chemistry, Nazarbayev University, 53 Kabanbay Batyr Ave., Astana 010000, Kazakhstan
* Correspondence: sadilov@nu.edu.kz (S.A.); mannix.balanay@nu.edu.kz (M.P.B.); Tel.: +7-717-270-6368 (S.A.); +7-717-269-4657 (M.P.B.)

Supplementary Information

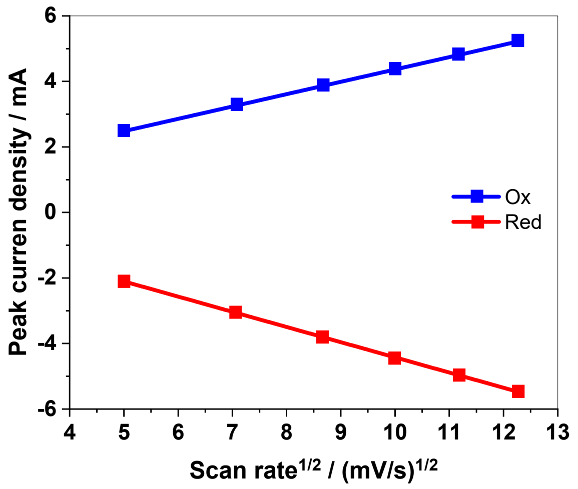

Figure S1. Correlation between Peak Current Density and Scan Rate for $Zn_{0.76}Co_{0.24}S$.

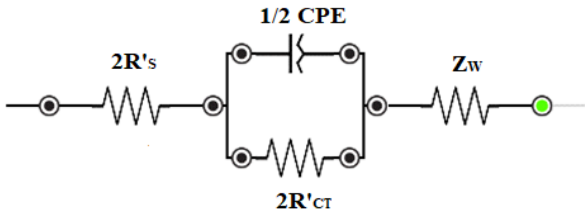

Figure S2. Equivalent electrical circuit used for fitting Nyquist plots of symmetrical dummy cells.

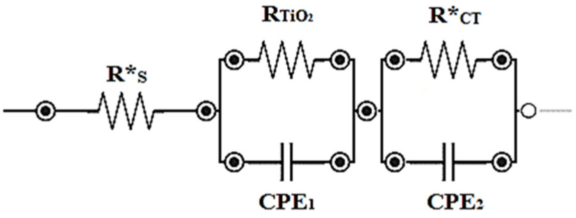

Figure S3. Equivalent electrical circuit used for fitting Nyquist plots of DSSCs.

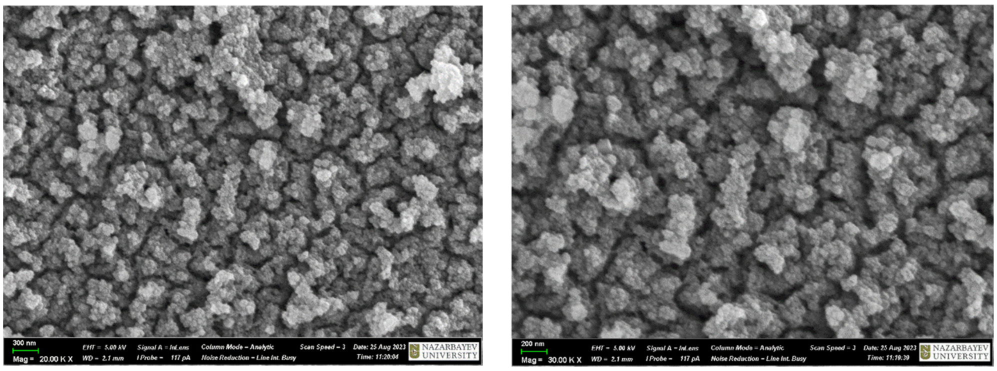

Figure S4. Scanning electron microscopy images of PB film deposited on FTO glass at different resolutions.
